# Tissue Clearing and Light Sheet Microscopy: Imaging the Unsectioned Adult Zebra Finch Brain at Cellular Resolution

**DOI:** 10.3389/fnana.2019.00013

**Published:** 2019-02-14

**Authors:** Mariana Diales Rocha, Daniel Normen Düring, Philipp Bethge, Fabian F. Voigt, Staffan Hildebrand, Fritjof Helmchen, Alexander Pfeifer, Richard Hans Robert Hahnloser, Manfred Gahr

**Affiliations:** ^1^Department of Behavioral Neurobiology, Max Planck Institute for Ornithology, Seewiesen, Germany; ^2^Institute of Neuroinformatics, University of Zurich/ETH Zurich, Zurich, Switzerland; ^3^Neuroscience Center Zurich (ZNZ), Zurich, Switzerland; ^4^Brain Research Institute, University of Zurich, Zurich, Switzerland; ^5^Institute of Pharmacology and Toxicology, University Hospital Bonn, University of Bonn, Bonn, Germany

**Keywords:** tissue clearing, light sheet microscopy, zebra finch, song system, large volume imaging

## Abstract

The inherent complexity of brain tissue, with brain cells intertwining locally and projecting to distant regions, has made three-dimensional visualization of intact brains a highly desirable but challenging task in neuroscience. The natural opaqueness of tissue has traditionally limited researchers to techniques short of single cell resolution such as computer tomography or magnetic resonance imaging. By contrast, techniques with single-cell resolution required mechanical slicing into thin sections, which entails tissue distortions that severely hinder accurate reconstruction of large volumes. Recent developments in tissue clearing and light sheet microscopy have made it possible to investigate large volumes at micrometer resolution. The value of tissue clearing has been shown in a variety of tissue types and animal models. However, its potential for examining the songbird brain remains unexplored. Songbirds are an established model system for the study of vocal learning and sensorimotor control. They share with humans the capacity to adapt vocalizations based on auditory input. Song learning and production are controlled in songbirds by the song system, which forms a network of interconnected discrete brain nuclei. Here, we use the CUBIC and iDISCO+ protocols for clearing adult songbird brain tissue. Combined with light sheet imaging, we show the potential of tissue clearing for the investigation of connectivity between song nuclei, as well as for neuroanatomy and brain vasculature studies.

## Introduction

Recent innovations in tissue clearing, combined with advances in light sheet microscopy and big data analysis, are bringing the exploration of whole brain three-dimensional space at single-cell resolution within our reach. Brain tissue is composed of high-refractive index (RI) molecules, lipids and proteins, embedded in a low RI medium, water. This RI mismatch leads to heterogeneity in light scattering, and consequently turns tissue opaque. Tissue clearing methods, which have flourished into several variants, generally try to reduce the RI mismatch by either substituting water by a higher RI medium or by removing or modifying the optical properties of the dry components (for review see Richardson and Lichtman, 2015).

Along with the available clearing protocols, the variety of species and tissue types examined has multiplied in recent years, encompassing varied species including mice, rats, rabbits, humans, zebrafish, *Xenopus*, octopus, fruit flies, and even the botanical model species *Arabidopsis* (Dodt et al., 2007; Palmer et al., 2015; Lee et al., 2016). As for avian tissue, tissue clearing has been commonly applied to the embryonic tissue of chicken (Botelho et al., 2017; Gómez-Gaviro et al., 2017), and more rarely to other avian embryos (Friocourt et al., 2017). Regarding songbird adult tissue, tissue clearing reports are limited to the zebra finch syrinx (Faunes et al., 2017) and the bengalese finch brain (Fujii et al., 2016).

Fujii et al. have, to our knowledge, published the only application of tissue clearing to the songbird brain. They applied the SeeDB (Ke et al., 2013) and *Clear*^*T*^ (Kuwajima et al., 2013) protocols to 1 mm-thick brain sections of both Bengalese finches (*Lonchura striata var. domestica*) and laboratory rats (*Rattus norvegicus*), and then compared the resulting transparency. Surprisingly, they showed limited success concerning the songbird brain tissue, as the finch samples became less transparent than the rat tissue, even though the finch sections were smaller in volume. Furthermore, no clearing of larger volumes of brain tissue was reported, and the capacity for imaging fluorescently labeled structures in the songbird brain remains unexplored. As of yet, the potential of optical clearing methods for research on the songbird brain remains surprisingly uncharted.

Songbirds are an important model system for the study of vocal learning because juvenile birds learn their songs via imitation of an adult tutor (for review see Bolhuis et al., 2010). Additionally, they are excellent models for studies of sensorimotor control and of neural plasticity, because singing requires precise motor coordination and their brains are highly responsive to sex hormones (for review, see Gahr, 2004).

The songbird brain organization shows distinct potential for the application of clearing techniques. Songbirds possess a dedicated brain circuit for song learning and production, the song system, which is organized as a network of discrete brain nuclei interconnected by brain-wide axonal projections, making three-dimensional explorations at cellular resolution particularly promising.

The exploration of the song system wiring has traditionally required segmentation into thin tissue sections. Whole-brain approaches have been limited to techniques lacking cellular resolution, such as CT or MRI (Poirier et al., 2008; Vellema et al., 2011). Interestingly, even though researchers have been trying to dissect the song system circuit since the 1970's (Nottebohm et al., 1976), new projections between song system nuclei and their afferents and efferents, and new projection neuron types have only recently been discovered (Akutagawa and Konishi, 2010; Roberts et al., 2017; Nicholson et al., 2018). Possibly, the loss of 3D information intrinsic to mechanical sectioning makes sparse projections difficult to detect.

We successfully clear and image unsectioned songbird forebrains. We chose the CUBIC protocol (Susaki et al., 2014, 2015) for its simplicity, low cost, low toxicity, potential compatibility with immunostaining, and good preservation of fluorescent proteins. We combine viral vector-driven fluorescent protein expression with light sheet microscopy to study projections between song system nuclei. We take a closer look at the projection between premotor nucleus HVC (proper name) and its efferent nucleus RA (robust nucleus of the arcopallium). Additionally, we also tested the iDISCO+ protocol (Renier et al., 2016) to compare the transparency obtained with that of a solvent-based method.

## Materials and Methods

### Animals

Zebra finches were reared in our breeding colonies in Seewiesen, Germany. Animal handling was carried out in accordance with the European Communities Council Directive 2010/63 EU and legislation of the state of Upper Bavaria. The government of Upper Bavaria, “Sachgebiet 54—Verbraucherschutz, Veterinärwesen, 80538 München” approved animal experiments (record number 55.2-1-54-2532-150-2016).

### Tissue Preparation

Four adult male zebra finches were intracranially injected into HVC using stereotactic coordinates, with either a lentivirus driving Tomato expression (*n* = 2, 2 weeks expression time—minimum expression time required to successfully label the HVC-RA projection), or an AAV driving GFP expression (*n* = 2, 3 weeks expression time), both under the control of the cytomegalovirus (CMV) promoter. Additionally, three adult male zebra finches were sacrificed (but not injected) to test the performance of the CUBIC protocol on a whole songbird brain (*n* = 1), as well as to provide tissue for the application of the iDISCO+ protocol (*n* = 2).

Birds were sacrificed by isoflurane overdose, perfused, and brains were extracted and post-fixed. Fixed brains where either kept whole or hemisected and trimmed to obtain forebrain hemispheres, because tissue clearing is known to perform better in smaller volumes. Subsequently, brain tissue clearing was carried out using either the CUBIC or iDISCO+ protocols (for further details see Supplementary Methods). We found that, with the CUBIC protocol, prolonging the immersion in reagent-1 solution to 10 days, instead of the 7 days recommended by the original protocol, helped improve final transparency.

### Imaging

Cleared tissue was transferred to the imaging solution and images were acquired using either the commercial Ultra Microscope II (LaVision), or the mesoSPIM system (www.mesospim.org). Post-processing of images was carried out using ImageJ (Schneider et al., 2012) and Imaris (Bitplane). For further details see Supplementary Methods.

During clearing progress, whole brain macroscopic images were acquired using a macroscope (Leica Z16 APO, 0.57x zoom). These images were then used to measure forebrain/whole brain maximal length in the rostro-caudal axis in order to obtain a rough estimate of tissue expansion/shrinkage.

## Results

### Tissue Clearing

**Figure 1:**
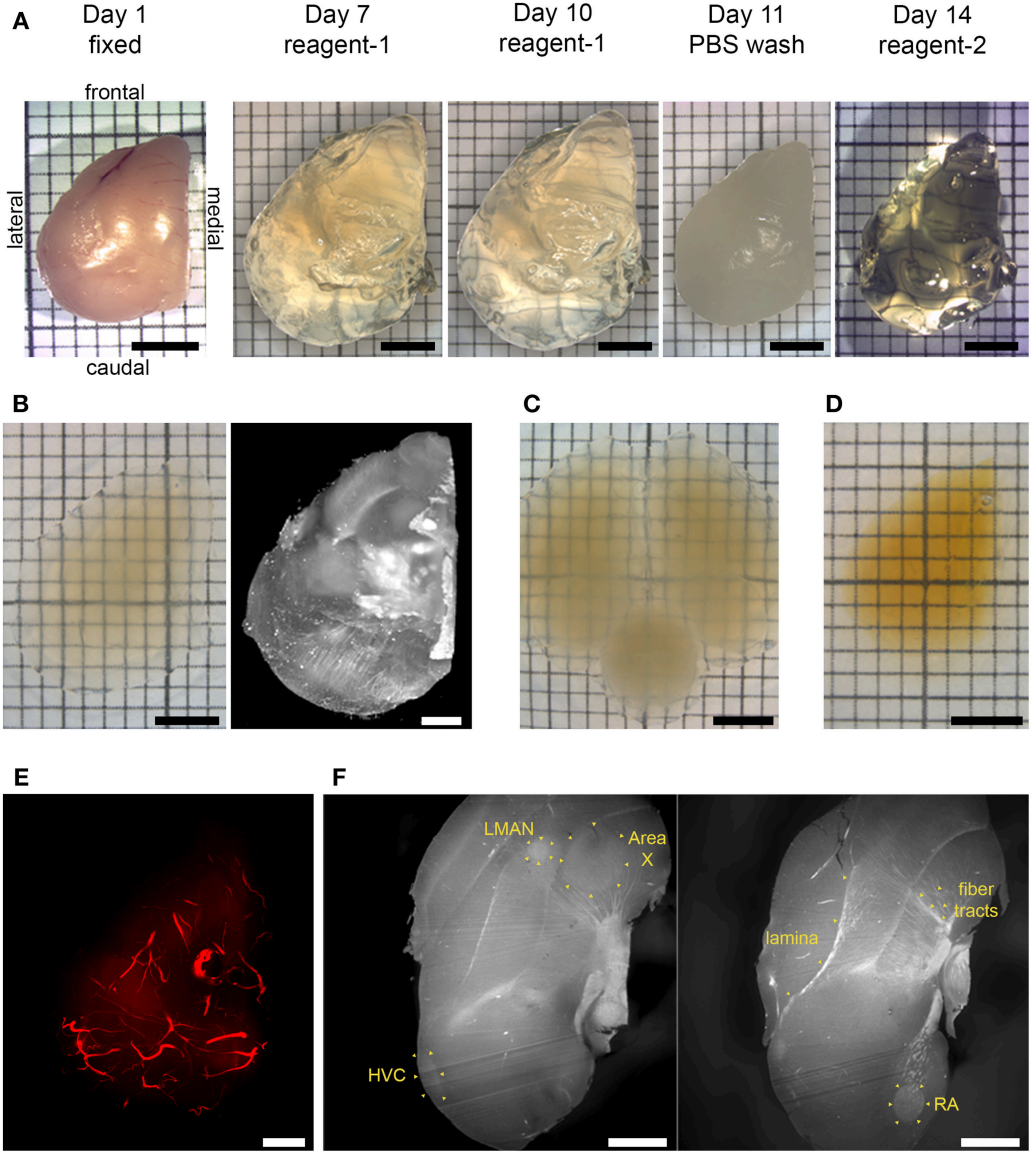
Chemical clearing of songbird brain tissue and visualization of autofluorescent features. **(A)** CUBIC pipeline. A fixed hemisphere was treated with reagent-1 to remove lipid components initially for 7 days, with reagent-1 immersion prolonged to a total of 10 days to increase transparency. Lipid removal was followed by a PBS wash, and then by reagent-2 immersion for refractive index (RI) adjustment for 3 days. **(B)** CUBIC-cleared hemisphere in imaging solution (left), and volume rendering resulting from light sheet imaging of the same hemisphere (right; mesoSPIM, 1.6x zoom, no emission filter). **(C)** CUBIC-cleared whole brain in imaging solution. **(D)** iDISCO+ cleared hemisphere in ethyl cinnamate. **(E)** Volume rendering of autofluorescent vasculature (mesoSPIM, 1.6x zoom). **(F)** Two sagittal optical sections showing autofluorescent anatomical landmarks, including laminae, myelinated fiber tracts, and song control nuclei HVC, RA, Area X, and LMAN (Ultra Microscope II, 1x zoom, 10 μm light sheet thickness; top is frontal, bottom is caudal). Scale bars in **(A,B)** left inset, **(C,D)** are 3 mm; in **(B)** right inset, **(E,F)** are 1 mm. **(A–E)** Dorsal view.

**Figure 2:**
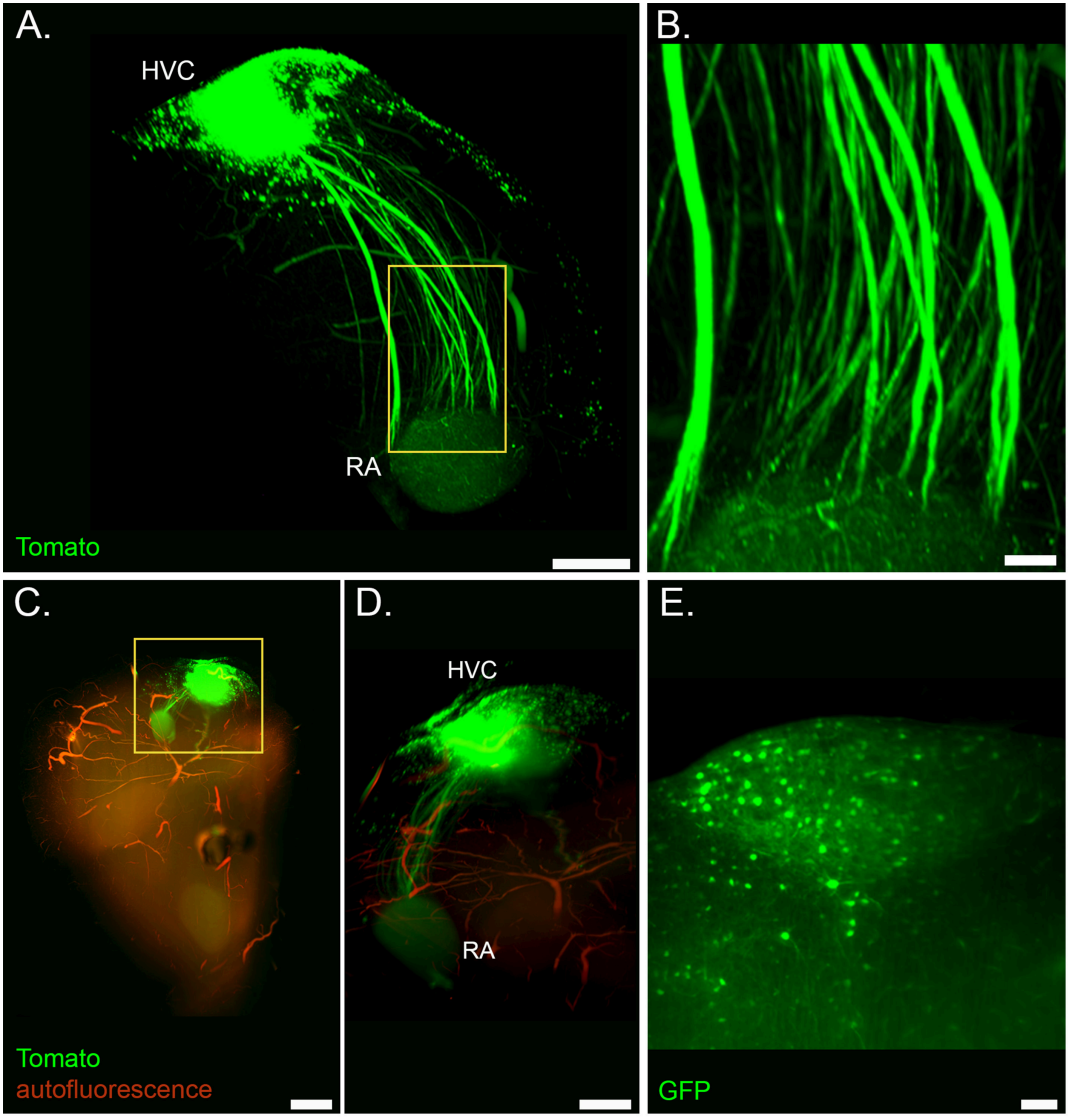
Light sheet microscopy of the cleared song system. **(A)** Volume rendering of the HVC-RA projection from a sample of a lentivirus injected bird (Ultra Microscope II, 1x zoom, frontal view). **(B)** Detail of axonal tracts from highlighted region in **(A)**. **(C)** Volume rendering of a whole forebrain hemisphere showing the labeled HVC-RA projection with overlaid autofluorescent signal from vasculature (same tissue sample as in **(A)**; dorsal view, top is caudal, bottom is frontal; mesoSPIM, 1.6x zoom) **(D)** Zoom into the highlighted region in **(C)**, showing the HVC-RA projection in more detail. **(E)** Optical section showing labeled cells in HVC from a sample of a AAV injected bird (Ultra Microscope II, 3x zoom, 10 μm light sheet thickness). Scale bars in **(A,D)** are 500 μm, in **(B,E)** 100 μm, in **(C)** 1 mm.

We successfully validated two tissue clearing methods for songbird brain tissue (Figure 1). We obtained transparencies comparable to those reported for CUBIC and iDISCO+ protocols, enabling single sided light sheet penetration in imaging volumes of least half an adult zebra finch forebrain (Figures 1, 2 and Supplementary Movie S1). We observed some expansion of CUBIC-cleared tissue, and some shrinkage for the iDISCO+ protocol (approximately 15% increase for CUBIC and 20% decrease for iDISCO+ in final maximal rostro-caudal length as compared to fixed tissue).

### Light Sheet Imaging

CUBIC tissue clearing enabled us to use light sheet imaging to visualize axonal tracts in the intact HVC-RA projection (Figures 2A–D and Supplementary Movie S1), as well as individual fluorescently-labeled cells throughout the entire HVC (Figure 2E).

Additionally, imaging of autofluorescent structures allowed visualization of cerebral vasculature throughout the forebrain in CUBIC-cleared tissue (Figures 1E, 2C,D), as well as anatomical landmarks in iDISCO+ cleared tissue (Figure 1F).

## Discussion

### Clearing the Songbird Brain

We successfully validated the application of tissue clearing for songbird brain tissue using the CUBIC and iDISCO+ protocols, and demonstrate that sufficient transparency can be achieved. The reason for the limited transparency achieved by Fujii et al. is still unclear. However, both clearing protocols they applied had been optimized for rodent tissue, and to our knowledge no clearing method has been previously optimized for songbird brain tissue. Possible explanations for unsatisfactory transparency in songbird brain tissue could be related to different chemical composition, and distinct anatomical organization, particularly of myelinated tissue (Karten et al., 2013). We found that prolonging the delipidation step in the CUBIC protocol helps achieve better final transparency. We also achieved good transparency using the iDISCO+ protocol, which we expected, because solvent-based protocols such as iDISCO+ provide the best clearing results. Nevertheless, with some exceptions (Schwarz et al., 2015), these protocols entail drawbacks such as poor fluorescent protein emission retention and the need for special dipping caps to protect objectives from harsh solvents.

### Clearing for Anatomical Landmarks and Vasculature

The intrinsic tissue fluorescence of cleared samples has previously been exploited for registering samples to a reference brain (Renier et al., 2016; Ye et al., 2016). Acquiring autofluorescence signals in a label-free channel can enable the creation of an average reference brain by pooling data from multiples samples. Subsequently, signals from labeled structures can be acquired in the appropriate channel and be aligned to the reference brain, enabling faster and more precise comparisons across treatment groups. IDISCO+ was specifically developed to optimize brain morphology preservation to facilitate automated registration of light sheet-imaged samples. Along with the chemical protocol, Renier et al. (2016) also developed ClearMap, an open-source software for automated mapping and analysis, including the registration of imaging data to custom reference atlases.

We show excellent preservation of brain morphology in iDISCO+ cleared songbird brain tissue (Figure 1F). Laminae, myelinated fiber tracts, and song system nuclei are all clearly visible. Anatomical landmarks in optical sections of light sheet-imaged forebrains show similar crispness to that of Nissl-stained thin tissue sections. Thus, clearing and light-sheet imaging show potential for the creation of songbird brain reference atlases.

CUBIC tissue clearing showed more limited preservation of major anatomical landmarks, but the autofluorescence of blood vessels enabled us to visualize vasculature throughout the songbird forebrain (Figures 1E, 2C,D). As the anatomy of major cerebral blood vessels is highly conserved across individuals (Xiong et al., 2017), with the bifurcation of the midsagittal sinus routinely used in various species including zebra finches as the zero-point for stereotactic works, it is not difficult to envision that such a reference point in vasculature could be exploited for the registration of CUBIC-cleared songbird brain tissue.

Furthermore, specific research questions on songbird brain plasticity could benefit from 3D analysis of cerebral vasculature and gross brain morphology; both brain region volume and microvasculature structure are key features frequently quantified in sex hormone studies (for review see Chen et al., 2013). Additionally, the anatomical landmarks we imaged in the iDISCO+ cleared tissue were comparable to those previously imaged in songbird brains through the use of MRI (Poirier et al., 2008; Vellema et al., 2011). Hence, these techniques could provide an alternative to MRI for studies of myelination and volumetric changes, with the added advantages of higher resolution and compatibility with targeted fluorescence imaging.

### Visualizing the Intact Song System at Cellular Resolution

Here, we focused on the projection from HVC to RA, as this is a known important projection within the song system. HVC sits at the apex of the song system and both HVC and RA are part of the two song system pathways, the anterior forebrain pathway for vocal learning and the motor pathway for song production. RA forms the output via its projection to motor neurons controlling the vocal organ. However, this is intended as a proof of principle, and we hope this pipeline will be used to study further pathways within the song system, as well as those connecting to its afferents and efferents.

In rodents, these techniques have already proven their value. 3D reconstructions of axonal pathways obtained from light sheet datasets of cleared tissue revealed previously undocumented projections and topographical features (Menegas et al., 2015; Renier et al., 2016; Ye et al., 2016). Likewise, a precise mapping of the wiring of the song system could improve our understanding on how auditory input entering the songbird brain is processed in order to produce the accurate motor output for imitating tutor song. Important contributions to this endeavor are already being fulfilled by the use of other promising techniques for precise 3D reconstructions at subcellular resolution, such as serial block-face light and electron microscopy (Oberti et al., 2011; Kornfeld et al., 2017) and expansion microscopy (Chen et al., 2015; for a first application of expansion light sheet microscopy to songbird brain tissue we refer to our other publication in the same research topic: Düring et al., 2019). Nonetheless, the main advantage of the combination of tissue clearing and light sheet microscopy is its simplicity and speed. Imaging an adult zebra finch forebrain hemisphere in one channel at sufficient resolution to follow axonal tracts is feasible in about 10 min. This makes these techniques excellent tools for investigations of long-range axonal projections within the songbird brain. Furthermore, projections in this complex system are known to respond to vocal learning experience and sex hormones. In combination with automated analysis tools, these techniques can make large-scale comparisons of long-rage axonal projections across different treatment groups feasible, and so enable more precise investigations into how this system rewires itself.

## Conclusion

We have shown the feasibility and discussed the potential applications of tissue clearing combined with light sheet microscopy for the visualization of intact long-range projections, vasculature, and anatomical landmarks in the songbird brain. We hope our findings will encourage the use of these techniques in order to advance our knowledge on the song system connectome, and lead to a better understanding on the anatomical basis of the neural mechanisms underlying vocal learning.

## Author Contributions

MR, DD, RH, and MG conceived of the study. MG, RH, and FH provided instruments, materials and reagents. MR performed all experiments. SH and AP developed the lentivirus. FV designed and built the mesoSPIM system. MR, DD, and PB performed the imaging. MR and DD interpreted the results and generated the figures. DD and MR wrote the first draft of the manuscript. All authors contributed to manuscript revision, read and approved the submitted version.

### Conflict of Interest Statement

The authors declare that the research was conducted in the absence of any commercial or financial relationships that could be construed as a potential conflict of interest. The reviewer MKS declared a shared affiliation, though no other collaboration, with several of the authors SH, AP to the handling Editor.

## Supplementary Material

Supplementary Movie S1 Intact HVC-RA projection in 3D. Animation of the 3D rendering of the HVC-RA projection labeled by CMV-tomato-lentivirus injection into HVC (Ultra Microscope II, 1x zoom; same as shown in Figure 2A).
