# Supplementary material for: Tissue Clearing and Light Sheet Microscopy: Imaging the Unsectioned Adult Zebra Finch Brain at Cellular Resolution
Source: Front Neuroanat. 2019 Feb 14;13:13. doi: 10.3389/fnana.2019.00013 (PMC6382697; doi:10.3389/fnana.2019.00013)
Supplement: Supplementary file 1 [file Data_Sheet_1.docx]

**Supplementary methods**

**Tissue preparation**

Zebra finches (*Taeniopygia guttata*) were sacrificed by isoflurane overdose, and perfused with 30 mL of 10 U/mL heparin in PBS, followed by 30 mL 4% paraformaldehyde (PFA) in PBS (pH 7.4, ice-cold). Brains were then extracted and post-fixed in 4% PFA at 4º C overnight.

**Tissue clearing**

Brain tissue (n=5) was cleared following the CUBIC protocol (Susaki et al., 2014, 2015). Briefly, reagent-1 (mixture of 25 wt% final concentration urea, 25 wt% final concentration Quadrol (Tetrakis (2-HP)) ethylenediamine), 15 wt% final concentration Triton X-100, and distilled water) and reagent-2 (mixture of 25 wt% final concentration urea, 50 wt% final concentration sucrose, 10 wt% final concentration triethanolamine, and distilled water) solutions were prepared and degassed. Fixed intact brains or forebrain hemispheres were first washed twice in PBS/0.01% (wt/vol) NaN_3_ at room temperature (RT) for at least 1h each, then immersed in 50% diluted reagent-1 solution at 37º C for 6h, and then transferred to undiluted reagent-1. Tissue was left in reagent-1 for 10 days at 37º C, with the solution being replaced every 2 days. We found that prolonging the immersion in reagent-1 solution (delipidation step) to 10 days, instead of the 7 days recommended by the original protocol, helped improve final transparency. At day 11, tissue was washed three times in PBS/0.01% (wt/vol) NaN_3_ at RT until the next day. Finally, samples were degassed, immersed in 50% diluted reagent-2 for 24h at RT, and transferred to undiluted reagent-2 for 2 days of incubation at 37º C (reagent-2 solution refreshed after first 24h).

Additionally, four fixed forebrain hemispheres (n=2) were cleared following the iDISCO+ protocol (Renier et al., 2016; latest protocol version from December 2016 obtained from the iDISCO official website, www.idisco.info), while omitting the use of antibodies.

Cleared brain tissue was transferred to the imaging solution (1:1 silicon oil and mineral oil mix for CUBIC-cleared tissue, ethyl cinnamate (Klingberg et al., 2017) for iDISCO+-cleared tissue) overnight before imaging.

For storage between imaging sessions, CUBIC-cleared samples were first washed with PBS/0.01% (wt/vol) NaN_3_ at RT for at least 1h, then immersed in 30% (wt/vol) sucrose in PBS/0.01% (wt/vol) NaN_3_ with shaking at RT overnight. When the samples sank to the bottom, they were immersed in O.C.T. compound (Tissue-Tek) and immediately stored at -80 °C.

To re-image, samples were first gradually thawed at RT, washed with PBS/0.01% (wt/vol) NaN_3_ at least twice for 1h each to remove sucrose and O.C.T. compound, and transferred to the imaging solution (1:1 silicon oil and mineral oil mix) overnight.

**Light sheet imaging**

Imaging was carried out in sagittal orientation (lateral side up) using either the commercially-available Ultra Microscope II (LaVision Biotec), or the mesoSPIM system (www.mesospim.org)

Cleared tissue (CUBIC-cleared: n=2 CMV-tomato-lentivirus injected birds, n=2 CMV-AVV-injected birds; and n=2 iDISCO+-cleared birds) was initially imaged on the Ultra Microscope II (LaVision Biotec) up to two weeks after the clearing process was completed. Samples were first glued (using standard super glue) to the sample holder, and inserted into a quartz imaging chamber filled with the corresponding imaging solution, where they were left for at least 1h to reduce imaging artifacts caused by air bubbles. The Ultra microscope II used was equipped with a bidirectional triple light sheet (laser lines: 488 and 561 nm), a 2x objective lens (Olympus MVPLAPO 2x), a 0.63x-6.3x zoom body (Olympus MVX-10 Zoom Body), and a scientific CMOS (sCMOS) camera (Andor Neo).

Imaging with the mesoSPIM (mesoscale selective plane illumination microscopy) system (n=2 CUBIC-cleared CMV-tomato-lentivirus injected birds) was carried out 4 weeks after the clearing process was completed. Samples were glued to a small weight and loaded into a quartz cuvette, then submerged in imaging solution (1:1 silicon oil and mineral oil mix) overnight, and imaged using a home-built microscope (mesoSPIM). The microscope consists of a dual-sided excitation path using a fiber-coupled multiline laser combiner (405, 488, 515, 561, 594, 647 nm, Omicron SOLE-6) and a detection path comprising an Olympus MVX-10 zoom macroscope with a 1x objective (Olympus MVPLAPO 1x), a filter wheel (Ludl 96A350), and a sCMOS camera (Hamamatsu Orca Flash 4.0 V3). For imaging Tomato, 561 nm excitation and a 561 longpass filter (RazorEdge LP 561 LP, AHF) were used, and for autofluorescence 647 nm excitation and a QuadLine Rejection band (ZET405/488/561/640, AHF). The excitation paths also contain galvo scanners (GCM-2280-1500, Citizen Chiba) for light-sheet generation and reduction of streaking artifacts due to absorption of the light-sheet. In addition, the beam waist is scanned using electrically tunable lenses (ETL, Optotune EL-16-40-5D-TC-L) synchronized with the rolling shutter of the sCMOS camera. This axially scanned light-sheet mode (ASLM) leads to a uniform axial resolution across the field-of-view (FOV) of 5-10 µm (depending on zoom & wavelength). Further technical details of the custom SPIM will be described elsewhere ([www.mesospim.org](http://www.mesospim.org/)).

**Image processing**

Images were processed using ImageJ (Schneider et al., 2012) and Imaris (Bitplane). ImageJ was used to combine z-stacks obtained from the acquisition of different channels from the same sample in the mesoSPIM. Imaris was used to generate figures 1 B right inset, E, and F, all panels in figure 2, and supplementray movie S1 (other than adjusting the histogram no further processing of the images was carried out).

**Supplementary bibliography**

Klingberg, A., Hasenberg, A., Ludwig-Portugall, I., Medyukhina, A., Männ, L., Brenzel, A., et al. (2017). Fully Automated Evaluation of Total Glomerular Number and Capillary Tuft Size in Nephritic Kidneys Using Lightsheet Microscopy. *J. Am. Soc. Nephrol.* 28, 452–459. doi:10.1681/ASN.2016020232.

Renier, N., Adams, E. L., Kirst, C., Wu, Z., Azevedo, R., Kohl, J., et al. (2016). Mapping of Brain Activity by Automated Volume Analysis of Immediate Early Genes. *Cell* 165, 1789–1802. doi:10.1016/j.cell.2016.05.007.

Schneider, C. A., Rasband, W. S., and Eliceiri, K. W. (2012). NIH Image to ImageJ: 25 years of image analysis. *Nat. Methods* 9, 671–5. Available at: http://www.ncbi.nlm.nih.gov/pubmed/22930834.

Susaki, E. a., Tainaka, K., Perrin, D., Kishino, F., Tawara, T., Watanabe, T. M., et al. (2014). Whole-brain imaging with single-cell resolution using chemical cocktails and computational analysis. *Cell* 157, 726–739. doi:10.1016/j.cell.2014.03.042.

Susaki, E. A., Tainaka, K., Perrin, D., Yukinaga, H., Kuno, A., and Ueda, H. R. (2015). Advanced CUBIC protocols for whole-brain and whole-body clearing and imaging. *Nat. Protoc.* 10, 1709–27. doi:10.1038/nprot.2015.085
